# Expressed sequence tags from the oomycete fish pathogen *Saprolegnia parasitica *reveal putative virulence factors

**DOI:** 10.1186/1471-2180-5-46

**Published:** 2005-08-02

**Authors:** Trudy Torto-Alalibo, Miaoying Tian, Kamal Gajendran, Mark E Waugh, Pieter van West, Sophien Kamoun

**Affiliations:** 1 Department of Plant Pathology, The Ohio State University, Ohio Agricultural Research and Development Center, Wooster, Ohio, USA; 2 National Center for Genome Resources, Santa Fe, New Mexico, USA; 3 Aberdeen Oomycete Group, College of Life Sciences and Medicine, University of Aberdeen, Foresterhill, Scotland, United Kingdom

## Abstract

**Background:**

The oomycete *Saprolegnia parasitica *is one of the most economically important fish pathogens. There is a dramatic recrudescence of *Saprolegnia *infections in aquaculture since the use of the toxic organic dye malachite green was banned in 2002. Little is known about the molecular mechanisms underlying pathogenicity in *S. parasitica *and other animal pathogenic oomycetes. In this study we used a genomics approach to gain a first insight into the transcriptome of *S. parasitica*.

**Results:**

We generated 1510 expressed sequence tags (ESTs) from a mycelial cDNA library of *S. parasitica*. A total of 1279 consensus sequences corresponding to 525944 base pairs were assembled. About half of the unigenes showed similarities to known protein sequences or motifs. The *S. parasitica *sequences tended to be relatively divergent from *Phytophthora *sequences. Based on the sequence alignments of 18 conserved proteins, the average amino acid identity between *S. parasitica *and three *Phytophthora *species was 77% compared to 93% within *Phytophthora*. Several *S. parasitica *cDNAs, such as those with similarity to fungal type I cellulose binding domain proteins, PAN/Apple module proteins, glycosyl hydrolases, proteases, as well as serine and cysteine protease inhibitors, were predicted to encode secreted proteins that could function in virulence. Some of these cDNAs were more similar to fungal proteins than to other eukaryotic proteins confirming that oomycetes and fungi share some virulence components despite their evolutionary distance

**Conclusion:**

We provide a first glimpse into the gene content of *S. parasitica*, a reemerging oomycete fish pathogen. These resources will greatly accelerate research on this important pathogen. The data is available online through the Oomycete Genomics Database [1].

## Background

Water molds such as *Saprolegnia *and *Aphanomyces *species are responsible for devastating infections on fish in aquaculture, fish farms and hobby fish tanks [2,3]. Members of the genus *Saprolegnia *cause saprolegniosis, a disease that is characterized by visible white or grey patches of filamentous mycelium on the body or fins of freshwater fish [2]. The oomycete *Saprolegnia parasitica *is economically one of the most important fish pathogens, especially on salmon and trout species. It causes tens of million dollar losses to aquaculture business worldwide, notably in Scotland, Scandinavia, Chile, Japan, Canada, and the USA [4,5]. *S. parasitica *infections are second only to bacterial diseases. In Japan, there is an annual mortality rate of 50% in coho salmon and elver due to *S. parasitica *infections [5-8]. In the United States, "winter kill" in catfish caused by *Saprolegnia *results in financial loses of up to 50%, which represents an economic loss of $40 million [6]. In Scotland, saprolegniosis also causes significant losses with the main problem occurring in salmon hatcheries.

Previously, *Saprolegnia *infections were kept under control with malachite green, an organic dye that is very efficient at killing the pathogen. However, since 2002 the use of malachite green has been banned around the world, due to its carcinogenic and toxicological effects. This has resulted in dramatic recrudescence of *Saprolegnia *infections. Therefore, there is an urgent need for novel alternative methods of management of Saprolegniosis.

*Saprolegnia *is often considered an opportunistic pathogen that is saprotrophic and necrotrophic [6]. However, it has become apparent that some *S. parasitica *strains are highly virulent and able to cause primary infections on salmon [3,9,10]. Infections occur on both eggs and fish. On eggs the disease is manifested by profuse mycelial growth on the egg surface resulting in rapid death. On fish, *Saprolegnia *invades epidermal tissues and can infect the entire surface of the body [11]. It causes cellular necrosis as well as dermal and epidermal damage, which ultimately leads to death by heamodilution [5,12]. Severe *Saprolegnia *infections result in lethargic behaviour, loss of equilibrium and commonly death of the fish [12,13].

Oomycete species can be pathogenic on plants, insects, crustaceans, fish, vertebrate animals, and various microorganisms [14,15]. Oomycetes, including *Saprolegnia*, have many fungus-like characteristics, but are not true fungi. A number of studies have indicated that they should be classified with the golden-brown algae and diatoms as stramenopiles [16-18]. This implies that oomycetes evolved genetic and biochemical mechanisms for interaction with animals and plants that are different from those of true fungi [14]. Indeed, oomycetes have several clearly defined developmental stages that are not found in fungal pathogens. For example, *Saprolegnia *species have a complex life cycle that includes both sexual and asexual reproduction. The asexual spore or sporangium is formed at the end of hyphal cells and can release many motile primary zoospores [6]. The primary zoospores swim only for a short time before they encyst and release a secondary zoospore. Secondary zoospores are motile for a longer period and are the main infection spore [5,11]. Secondary zoospores are able to encyst and release new zoospores several times. This process is called "polyplanetism" [19], and may have evolved to allow the zoospores to have several attempts to locate and infect a host [6]. Uniquely within the class of oomycetes, secondary zoospores of *Saprolegnia *can possess hairs that are thought to be required for attachment to the host [11,19]. For example zoospores of *S. parasitica *have long hooked hairs that are believed to increase efficiency of the attachment to the fish hosts [12,19].

Although different in their selection of host organisms, plant and fish pathogenic oomycetes have many features in common. Evidently, the formation of specialised spore structures including zoospores, sporangia and oospores are similar. Also, infection strategies are comparable to some extent, involving encystment and attachment of zoospores on host surfaces, and penetration of host tissues. Furthermore, it is hypothesized that similar to biotrophic plant pathogenic oomycetes, such as *Peronospora *and several *Phytophthora *species, suppression of host defenses is likely to play a critical role in *Saprolegnia *pathogenesis. Host defense suppression by oomycetes remains poorly understood and only a few pathogen molecules that suppress host defenses have been identified in pathogenic oomycetes [20-22]. There is intriguing evidence that *Saprolegnia*-infected fish appear to be immuno-compromised [23,24]. Possibly, virulence factors secreted by the pathogen might account for the immuno-suppression and the lack of an effective response to pathogen infection.

Despite the huge economic importance of animal pathogenic oomycetes, such as *S. parasitica*, very little is known about the fundamental molecular mechanisms underlying development, pathogenicity and host specificity [14]. A thorough understanding of the basic molecular processes in *Saprolegnia*, the nature of the interactions with its hosts, and the identification of genes and proteins involved in these processes, could lead to novel control strategies that increase fish health, reduce disease losses and increase profits. In this study we used a genomics approach to gain a first insight into the transcriptome of *S. parasitica*. We generated random cDNA sequences (expressed sequence tags or ESTs) from a cDNA library of *S. parasitica *to identify genes that inform us about the biology of this organism and that could be involved in pathogenicity. We provide an overview of the identified sequences as well as a detailed description of a number of notable cDNAs. The data is available through a publicly accessible website as part of the Oomycete Genomics Database (OGD) [1].

## Results

### cDNA library and sequencing

We constructed a unidirectional cDNA library using mRNA isolated from mycelium of ATCC90214, a *S. parasitica *strain isolated from diseased salmon [25]. Mycelium was obtained from nutrient deprived 29-day-old *in vitro *culture. In other oomycetes, such as *P. infestans*, similar treatments promote the expression of stress-related genes and possibly mimic infection conditions [26,27]. A total of 2296 sequencing reactions corresponding to the 5' end of the cDNA insert were performed. Of these, 2102 gave readable sequences. The sequences and the quality (phred) scores were fed into NCGR's X Genome Initiative (XGI) [28] annotation pipeline and subjected to further quality controls [29]. 1510 ESTs remained after vector and low quality sequences were removed. Of these, 5% were assessed to be in reverse orientation based on the occurrence of at least eight consecutive A residues within the first 38 bp. Following additional quality screening and assembly, 1279 consensus sequences (so-called unigenes) were obtained consisting of 1146 singletons and 133 consensus with two or more ESTs. In total, 525,944 bp of assembled sequences were obtained corresponding to an average consensus sequence length of 411 bp. At 61%, the GC content of the assembled sequences was relatively high and similar to the GC content reported for *Phytophthora *spp. (57–58%) [14,30].

### Sequence annotation

The 1279 consensus sequences were annotated using the methods implemented in the XGI pipeline (see methods). A total of 609 sequences (48%) showed significant similarities to known protein sequences (E value < 10^-5^) based on BLASTX searches, 398 (31%) gave significant hits to protein motifs in the BLOCKS+ database, and 600 (47%) gave hits to the InterPro protein motif database with at least one of the 12 algorithms implemented in InterProScan. Among these, InterPro database searches with HmmPfam revealed 340 hits. In total, 585 consensus sequences (46%) could be assigned identities based on the Gene Ontology (GO) Consortium (see methods). The differences between the different analyses are expected for such bioinformatics annotations and reflect, among other things, differences in sensitivity between the various programs. A total of 70 sequences were positive with the PexFinder algorithm and are candidate for carrying signal peptides and encoding extracellular proteins.

### Taxonomic identity of the homologs of *S. parasitica *cDNAs

Considering the classification of oomycetes as stramenopiles, it was interesting to systematically examine the taxonomic identity of the homologs of *S. parasitica *cDNAs. To this end, we took advantage of the availability of several eukaryotic genomes to compile a data set of 270334 proteins covering six major phyla of eukaryotes: fungi, animals, plants, alveolates, discicristates, and heterokonts. The data included the complete proteomes of at least one species for all phyla except for discicristates (see methods). We used BLASTX to compare the 1279 *S. parasitica *unigenes to these eukaryotic proteins. In total, 715 sequences showed no significant hits (E value > e-5). Of the 582 sequences that showed significant hits, 32% (185) had the top hit to a diatom protein confirming the affinity between diatoms and oomycetes as stramenopiles. In contrast, only 56 (about 10%) sequences had a fungal protein as a top hit.

### Phylogenetic analyses

**Figure 1:**
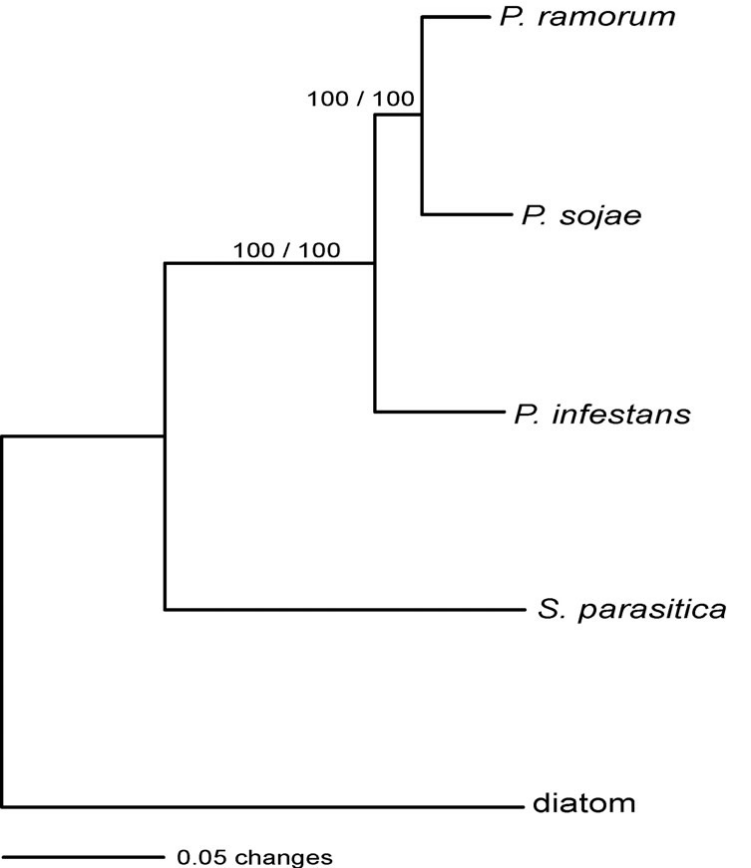
Phylogenetic relationships between *Saprolegnia parasitica *and four other stramenopiles. The phylogenetic tree was constructed using the neighbor joining method based on concatenated alignments from 18 conserved proteins (2533 amino acids). Percentile bootstrap values based on 1000 replications and obtained with the neighbor joining methods/ maximum parsimony methods are indicated at the nodes. The scale bar represents 5% weighted amino acid sequence divergence.

We also exploited the sequence data to examine phylogenetic affinities between *S. parasitica*, three *Phytophthora *spp., and the diatom *Thalassiosira pseudonana*. We performed reciprocal BLAST searches to identify a common set of conserved protein sequences between the five species. Multiple alignments of the conserved portions of 18 different proteins covering 2533 amino acids were concatenated and used in phylogenetic analyses. The obtained tree clearly supported a monophyletic relationship between the four oomycetes, and consistent with published phylogenies of *Phytophthora *[31,32] suggested that *P. sojae *and *P. ramorum *are more closely related (Fig. 1). Average amino acid identity among sequences of the *Phytophthora *spp. was 93% (range, 91.1–95.0%). In contrast, average amino acid identity between *S. parasitica *and the three *Phytophthora *spp. was 77% (range, 76.4–77.9%). Average amino acid identity between the diatom and the four oomycetes was 66.7% (range, 66.2–67.5%).

### Most represented protein domains

As described above, searches of the InterPro database with HmmPfam revealed 340 hits among the 1297 consensus sequences. We classified the InterPro domains based on their abundance (Table 1). The 340 hits corresponded to 198 different InterPro domains. Of these, 29 domains were represented three times or more (range, 3–22). By far the most abundant domain was IPR000719 for eukaryotic protein kinase, which was represented 22 times. Other domains that function in signal transduction, such as ankyrin-repeat, G-protein, Ras GTPase, myb DNA binding, were also relatively abundant. Other well-represented domains comprised fungal type I cellulose-binding domain (IPR000254), PAN domain (IPR003014), and papain cysteine protease family (IPR000668).

### Fungal type I cellulose binding domain

**Figure 2:**
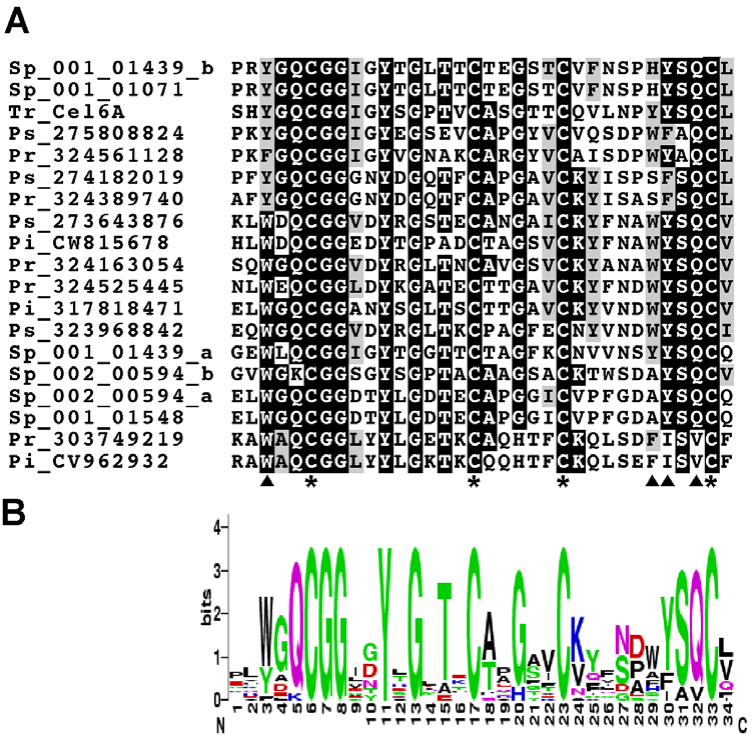
Fungal type I cellulose binding domain (CBD) is widespread in oomycetes. (A) Multiple sequence alignment of 18 oomycete type I CBDs with the domain of *Trichoderma resei *Cel6A (Tr_Cel6A). The four conserved cysteine residues are marked with asterisks. The glutamine and three aromatic residues that are known to be important for binding the carbohydrate substrate are shown by arrows. Sequence names refer to the *Saprolegnia parasitica *unigene (Sp_), *Phytophthora infestans *(Pi_), *Phytophthora sojae *(Ps_), and *Phytophthora ramorum *(Pr_) followed by the OGD accession number, GenBank accession number, or NCBI Trace Archive identifier (Ti number). Multiple domains originating from the same sequence are marked with the letters a or b at the end of the sequence name. (B) Consensus sequence pattern of the oomycete type I CBD. Consensus sequence was calculated using WebLogo. The bigger the letter, the more conserved the amino acid site. The positions of amino acids in the consensus sequence correspond to the positions in the sequence alignment in panel A.

Four sequences showed similarities to fungal-type I cellulose binding domain (CBD) (InterPro domain IPB000254). Three of these occurred in cDNAs predicted to encode extracellular proteins. Two cDNAs, Sp_002_00594 and Sp_001_01439, encoded putative proteins with two CBDs. The full length sequence of cDNA SPM5F8 (Sp_001_01439) was obtained (GenBank accession number DQ143887). This cDNA contained an ORF of 306 bp corresponding to a protein of 101 amino acids. SignalP [33] analysis of the predicted protein identified a 20-amino acid signal peptide with a significant mean S value of 0.93. Domain IPB000254 has been mainly reported in fungi [34], but also occurs in one protein from *Ectocarpus siliculosus *Virus EsV-1 (*Phycodnaviridae*), a viral pathogen of brown algae [35]. To determine the extent to which the CBD occurs in oomycetes, we performed iterative BLAST searches of all publicly available *Phytophthora *sequences using the *S. parasitica *CBD sequences. In total, 12 different sequences similar to the *S. parasitica *CBDs were recovered from *P. infestans *(3), *P. sojae *(4), and *P. ramorum *(5). The 18 oomycete CBD sequences aligned perfectly over a 34 amino acid region. Multiple alignments of the oomycete CBDs with the well-studied CBD of cellobiohydrolase I (Cel6A) of *Trichoderma resei *[36,37] suggested that the major features of the domain are conserved in oomycetes (Fig. 2). Amino acid residues defining the Cel6A domain including the four cysteine backbone, as well as a glutamine (Gln32) and the three tyrosines (Tyr3, Tyr29 and Tyr30) that are important for binding to cellulose, were frequently conserved. Nonetheless, tyrosines, particularly Tyr3 and Tyr29, were often replaced by other aromatic residues, such as tryptophane and phenylalanine, as observed for various fungal CBDs, such as endoglucanase I of *T. resei *[38].

### CBEL-like sequences

**Figure 3:**
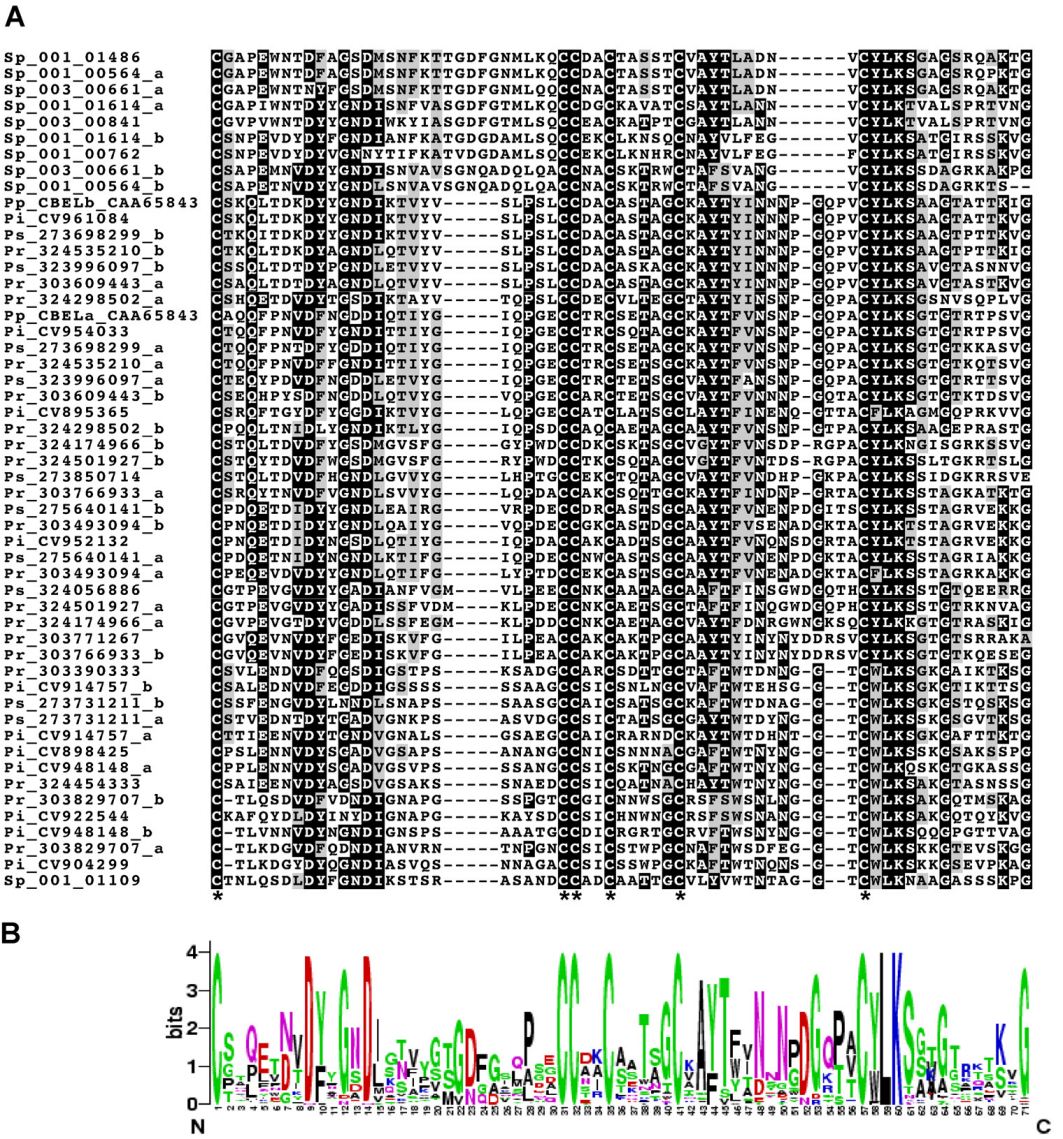
PAN module/Apple domain is widespread in oomycetes. (A) Multiple sequence alignment of 52 oomycete PAN module/Apple domain including the two domains of the previously described CBEL protein of *Phytophthora parasitica *(Pp_CBELa and Pp_CBELb). The six conserved cysteine residues are marked with asterisks. Sequence names refer to the *Saprolegnia parasitica *unigene (Sp_), *Phytophthora infestans *(Pi_), *Phytophthora sojae *(Ps_), and *Phytophthora ramorum *(Pr_) followed by the OGD accession number, GenBank accession number, or NCBI Trace Archive identifier (Ti number). Multiple domains originating from the same sequence are marked with the letters a or b at the end of the sequence name. (B) Consensus sequence pattern of the oomycete oomycete PAN module/Apple domain. Consensus sequence was calculated using WebLogo. The bigger the letter, the more conserved the amino acid site. The positions of amino acids in the consensus sequence correspond to the positions in the sequence alignment in panel A.

Seven distinct cDNA sequences showed similarity to the Cellulose Binding, Elicitor and Lectin-like protein (CBEL), a 34-kDa extracellular glycoprotein that was first isolated from *Phytophthora parasitica *[39,40]. Five of these CBEL-like cDNAs were also predicted to encode extracellular proteins based on PexFinder analyses. CBEL has a dual function: (1) it is required for attachment to plant surfaces, (2) it elicits necrosis and defense gene expression in tobacco plants [39,40]. CBEL contains two regions with similarity to the PAN module (InterPro: IPR000177), a conserved domain that includes the Apple domain and functions in protein-protein or protein-carbohydrate interactions [41]. The similarity to CBEL in the identified *S. parasitica *cDNAs centered on the PAN module regions. Three of the seven *S. parasitica *cDNAs contained two PAN-like domains while the other four had a single domain. We used these sequences to survey the available *Phytophthora *sequences for additional PAN/CBEL-like domains using iterative BLAST searches. In total, 42 PAN/CBEL-like domains were identified in 28 putative proteins of *P. infestans*, *P. sojae*, *P. ramorum*, and *P. parasitica *suggesting that this domain is widely distributed in oomycetes (Fig. 3). Multiple alignment of the 52 oomycete CBEL-like domains revealed a conserved pattern centered around a conserved core of six cysteines (Fig. 3B).

### Glycosyl hydrolases

Six cDNAs showed similarity to various classes of glucanases. One of these, Sp_001_01488, showed significant similarity to microbial endo-1,3-β-glucanases (glycosyl hydrolase family 17), as well as high similarity to the recently described gene, *piendo1*, from *P. infestans *[42]. The 1258 bp insert of a full-length cDNA (SPM16A2) corresponding to this endo-1,3-β-glucanase was fully sequenced (GenBank accession number AY974332). The sequence revealed a single ORF of 1197 bp encoding a predicted protein of 398 amino acids with 38% identity to PIENDO1. SignalP [33] analysis of the predicted protein identified a 19-amino acid signal peptide with a significant mean S value of 0.94. Previously, phylogenetic analyses of several *Phytophthora *hydrolytic enzymes, including PIENDO1, revealed unexpected affinity to fungal proteins [27,42-44]. Indeed, BLASTP searches of the protein encoded by SPM16A2 showed significant similarities to fungal and bacterial proteins (top hits with E value = 2e-21 and 3e-22, respectively) but none to other eukaryotic proteins.

### Proteases

We found a set of 12 cDNAs with similarity to aspartyl (2), serine (3), and cysteine (7) proteases among the annotated sequences of *S. parasitica*. The sequence of SPM3B2, a full length cDNA corresponding to unigene Sp_004_00851 was obtained (GenBank accession number AY974331). This cDNA encoded a putative protein of 379 amino acids. BLASTP searches of the MEROPS database [45] revealed significant similarity to pepsin aspartic proteases such as cathepsin D (MEROPS Family A01, E value = 1e-72 for best hit). SignalP [33] analysis of the predicted protein identified a 17-amino acid signal peptide with a significant mean S value of 0.73. We also determined the full length sequence of cDNA SPM9F1 (Sp_001_01152) (GenBank accession number AY974330). This cDNA encoded a putative protein of 524 amino acids with significant similarity to papain cysteine proteases (MEROPS Family C01A, E value = 1e-58 for best hit). SignalP [33] analysis of the predicted protein identified a 22-amino acid signal peptide with a significant mean S value of 0.75. BLASTP searches against GenBank NR and the *Phytophthora *data sets revealed that both proteases are widely distributed among eukaryotes and oomycetes.

### Protease inhibitors

**Figure 4:**
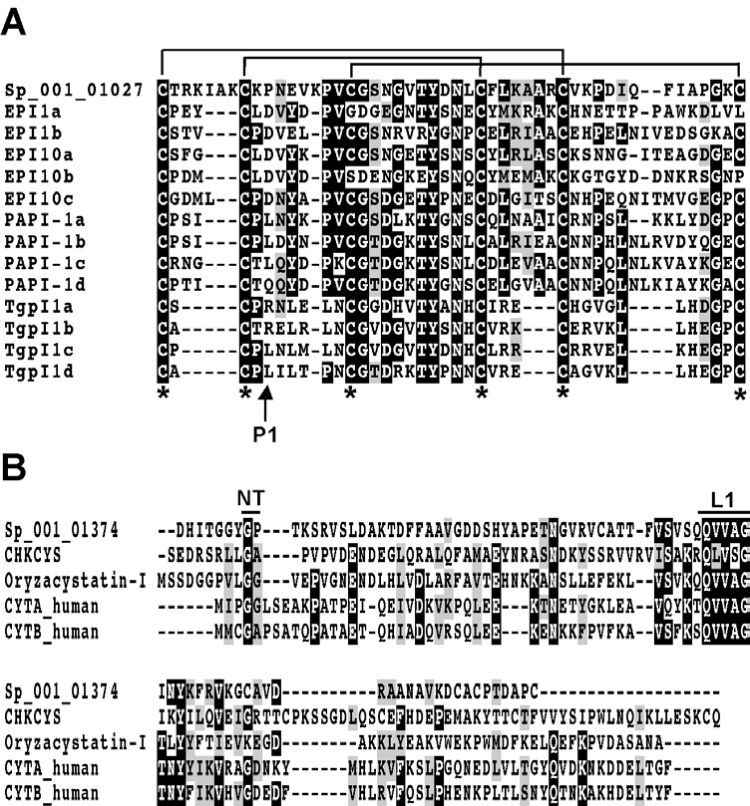
Protease inhibitors in *Saprolegnia parasitica*. (A) Sequence alignment of Sp_001_01027 predicted amino acid sequence with representative Kazal family inhibitor domains. Protein names correspond to protease inhibitors of *Saprolegnia parasitica *Sp_001_01027, *Phytophthora infestans *EPI1 (EPI1a-b, AY586273) and EPI10 (EPI10a-c, AY586282), the crayfish *Pacifastacus leniusculus *(PAPI-1a-d, CAA56043), and the apicomplexan *Toxoplasma gondii *(TgPI-1a-d, AF121778). The conserved cysteine residues that define the Kazal family protease inhibitor domain are marked with asterisks. The putative disulfide linkages formed by cysteine residues within the predicted Kazal domains are shown. The position of the predicted P1 residues is shown by an arrow. (B) Sequence alignment of *Saprolegnia parasitica *Sp_001_01374 (N-terminal fragment of the mature protein), chicken egg white cystatin (CHKCYS, P01038, mature protein), rice oryzacystatin-I (Oryzacystatin-I, P09229), human cystatin A (CYTA_human, P01040) and cystatin B (CYTB_human, P04080). The proposed active-site residues in cystatins, forming the N-terminal trunk (NT) and first binding loop (L1), are indicated.

We have also identified two *S. parasitica *cDNAs with similarity to protease inhibitor domains of two structural classes: (1) Kazal-like serine protease inhibitor (InterPro domain IPR002350, MEROPS family I1), (2) cysteine protease inhibitor (InterPro IPR000010, MEROPS family I25). We further analyzed these two cDNAs by aligning their putative inhibitor domains to those of known protease inhibitors (Fig. 4). Sp_001_01027 showed significant similarity to the Kazal-like inhibitors recently described by Tian *et al*. [21] from *P. infestans *and other plant pathogenic oomycetes. Amino acid residues defining the Kazal motif, including the six cysteine backbone, tyrosine and asparagine residues, were conserved in Sp_001_01027 (Fig. 4A). The predicted active site P1, which is central to the specificity of Kazal inhibitors [46,47], consisted of a proline, and therefore differed from all previously reported oomycete Kazal domains [21]. Sp_001_01374 is predicted to encode a secreted protein that bears the hallmark of the cystatin class of cysteine protease inhibitors including the highly conserved QXVXG motif in the first binding loop (L1) [48] (Fig. 4B). These findings suggest that secretion of protease inhibitors is a common feature of oomycetes.

### Thiamine biosynthetic enzyme

We identified one sequence (Sp_001_00801) with significant similarity to a thiamine biosynthetic enzyme from plants (top hit to protein AAV92556 from the conifer *Pseudotsuga menziesii*, E value = 2e-63) and fungi (*Schizosaccharomyces pombe *protein CAA21093, E value = 3e-53). Unlike *S. parasitica *and other oomycetes, members of the genus *Phytophthora *are thiamine auxotrophs, they require exogenous sources of thiamine for growth [49,50]. Interestingly, BLAST searches of the genome sequence reads of *P. sojae *and *P. ramorum*, as well as all available sequences of *P. infestans*, failed to reveal sequences with similarity to Sp_001_00801 or to the plant and fungal enzymes. These findings suggest that this thiamine biosynthetic enzyme may have been lost in the *Phytophthora *lineage and could be related to thiamine auxotrophy in this genus.

## Discussion

In this study we generated 1510 high quality ESTs from *S. parasitica*, an economically important and reemerging oomycete pathogen that causes multimillion dollar losses in the aquaculture industry. The ESTs were generated from a cDNA library constructed from one-month old nutrient deprived mycelium cultures. So far significant data sets of oomycete ESTs have been described for three plant pathogenic species, *P. infestans *[26,27], *P. sojae *[30] and *P. parasitica *[51]. Therefore, the *S. parasitica *ESTs offer some insights into the transcriptome of animal pathogenic oomycetes, which have been extremely understudied. Prior to this work, only 13 nucleotide and 2 protein sequences of *S. parasitica *could be retrieved from GenBank (March 2005 release). The sequence data and the corresponding annotations described in this study are accessible through an interactive public resource, the Oomycete Genomics Database (OGD). We hope that this pilot genomics project will accelerate research on this important pathogen and lays the foundation for more significant genome and cDNA sequencing initiatives of animal pathogenic oomycetes.

We used the *S. parasitica *ESTs to confirm the phylogenetic affinities between oomycetes and diatoms [16-18]. About 32% of the *S. parasitica *sequences that showed significant similarities to eukaryotic proteins matched a protein of the diatom *T. pseudonana *as a top hit. Within the oomycetes, *S. parasitica *is classified with other water molds, such as *Achlya *and *Aphanomyces*, in the order Saprolegniales [52-54]. These species are morphologically very distinct from the great majority of plant pathogens, such as the Peronosporales *Phytophthora *and downy mildews, or the Pythiales *Pythium *[55]. Indeed, the *S. parasitica *sequences tended to be relatively divergent from *Phytophthora *sequences. For example, based on the sequence alignments of 18 different conserved proteins, the average amino acid identity between *S. parasitica *and three *Phytophthora *spp., *P. infestans*, *P. sojae*, and *P. ramorum*, was 77% compared to 93% within *Phytophthora*. Differences in transcript content were also noted. cDNAs with similarity to elicitins, a group of 10-kDa proteins that occur in all *Phytophthora *and some *Pythium *species [56-61] and form 1–2% of mycelial ESTs in *Phytophthora *[26,27,62], were not found in the *S. parasitica *dataset. Although, elicitin-like genes could very well occur in the genome of *S. parasitica*, they do not seem to be abundantly expressed in mycelium. Elicitins were shown to function as sterol carriers [63,64]. Among the oomycetes, members of the Saprolegniales are able to synthesize sterols de novo whereas *Phytophthora *and *Pythium *spp. are sterol auxotrophs [50]. Possibly, *S. parasitica *may not require sterol carriers, such as elicitins, for optimal hyphal growth. Another difference between *Phytophthora *and *Saprolegnia *involves thiamine metabolism. Members of the genus *Phytophthora *are thiamine auxotrophs and require exogenous sources of thiamine for growth [49,50]. We identified one sequence in *S. parasitica *(Sp_001_00801) that shows significant similarity to a thiamine biosynthetic enzyme from plants and fungi but that is absent in the draft genome sequences of *P. sojae *and *P. ramorum*. This finding suggests that this thiamine biosynthetic enzyme may have been lost in the *Phytophthora *lineage and could be related to thiamine auxotrophy in this genus.

We searched the annotated data set for *S. parasitica *sequences that show similarities to known proteins and protein motifs that could inform us about the biology and pathology of this microbe. About half of the unigenes showed similarities to known protein sequences and could be assigned a putative function. A number of sequences showed particularly interesting similarities. cDNAs with similarity to signal transduction proteins, such as kinases and transcription factors, were particularly abundant. In total, 70 cDNAs encoded proteins with a putative signal peptide that are potentially secreted to the extracellular space. Secretion is an essential mechanism for delivery of virulence factors by eukaryotic pathogens to their appropriate site in infected host tissue. Therefore, several putative secreted proteins of *S. parasitica*, such as CBD proteins, CBEL-like proteins, glycosyl hydrolases, proteases, and protease inhibitors could function in virulence and will be worthy of additional studies.

Phylogenetic analyses indicated that several *Phytophthora *proteins, particularly hydrolytic enzymes such endopolygalacturonases, pectate lyases, exo-1,3-beta-glucanases, and an endo-1,3-beta-glucanase, are more similar to fungal proteins than to their counterparts in other eukaryotes [27,42-44]. These observations are in sharp contrast with phylogenies constructed from ribosomal sequences or compiled protein sequences from mitochondrial and housekeeping chromosomal genes, which indicate considerable evolutionary distance between oomycetes and fungi [16-18,65,66]. The apparent discrepancies between these phylogenies could reflect convergent evolution in the arsenal of hydrolytic enzymes between these pathogens, perhaps as a result of common mechanisms of infection among filamentous microbes [27,67]. The *S. parasitica *sequences allowed us to evaluate whether the similarity to fungal proteins extends to oomycetes other than *Phytophthora *and to animal pathogenic oomycetes. Although no *S. parasitica *sequences similar to the cell wall degrading enzymes endopolygalacturonases and pectate lyases were found, we identified a cDNA, SPM9F1, that encodes a 524 amino acid protein with high similarity to endo-1,3-β-glucanases, including the recently described PIENDO1 of *P. infestans *[42]. Similar to PIENDO1, SPM9F1 was most similar to fungal glucanases and no significant BLASTP hits (E value > 0.01) were observed to non-fungal eukaryotic proteins. Therefore, conservation in the arsenal of hydrolytic enzymes appears to extend beyond *Phytophthora *spp. to the Saprolegniales and animal pathogenic oomycetes.

Domain annotation of the *S. parasitica *sequences revealed the occurrence of a protein domain typically associated with fungi. Type I CBDs (InterPro domain IPB000254) are thought to be unique to fungi [34], although a related domain also occurs in the brown algae viral pathogen *Ectocarpus siliculosus *Virus EsV-1 (*Phycodnaviridae*) [35,68]. In this study, we found that this domain is widespread and diverse in *S. parasitica *and other oomycetes. A total of 18 domains from four oomycete species were found to share a 34 amino acid region that aligns perfectly with the canonical *T. resei *Cel6A CBD highlighting a core of conserved four cysteines and aromatic residues known to bind the cellulosic substrate [36,38]. Interestingly, the occurrence of this CBD in a virus of brown algae, which are related to oomycetes, suggests that type I CBDs might be more widespread in stramenopiles although we did not detect them in the draft genome sequence of the diatom *T. pseudonana*. In fungi, the CBDs are usually located in the N- or C-terminal regions of hydrolytic enzymes, such as cellulases and xylanases, and function by concentrating the catalytic domains on the surface of the insoluble cellulose substrate [34]. One of the *S. parasitica *cDNAs, SPM5F8, encodes a small 101 amino acid protein with two CBDs. Such a protein could function as a scaffolding component of the multienzyme complex known as cellulosome [34]. The function of this and other CBD proteins in *S. parasitica *may relate to attachment to organic debris on the host surface or during saprophytic growth. Alternatively, since cellulose is a major component of the cell wall of oomycetes, these proteins may play endogenous function in cell wall biogenesis.

Seven *S. parasitica *cDNAs showed similarity to CBEL, a 34-kDa cell wall glycoprotein of *P. parasitica *that binds to cellulose and host surfaces, functions in the agglutination of red blood cells, and elicits necrosis and defense gene expression in tobacco [39,40]. The similarity centered mainly on two regions of CBEL that match the PAN module/Apple domain (InterPro IPR000177). The CBEL-like PAN module, which is thought to function in protein-protein or protein-carbohydrate interactions [41], appeared to be particularly diverse in oomycetes with 52 different sequences identified in five species. The PAN module was found in proteins with diverse functions, such as the blood coagulation factor XI and the plasma protein pre-kallikrein [41]. Recently, several secreted proteins from apicomplexan mammalian parasites were found to contain Apple-like domains and are thought to play a role during parasite attachment and invasion of host cells [69-72]. For example, MIC4, an adhesin secreted by the apicomplexan *Toxoplasma gondi*, contains six Apple domains [69]. It remains to be determined the extent to which the secreted PAN/CBEL-like proteins of *S. parasitica *play a role in attachment and invasion during interaction with the fish host. Nonetheless, it appears that in oomycetes, similar to the apicomplexan parasites, some adhesins are secreted PAN module proteins.

Proteolytic enzymes are considered important virulence factors that aid in host colonization and release of nutrients by animal pathogenic microbes. It has long been known that the *Saprolegnia *spp. pathogenic on fish exhibit significant extracellular protease activity and it was postulated that this enzymatic activity contributes to pathogenesis [73]. A serine protease gene, *AaSP2 *from the related crayfish pathogen *Aphanomyces astacus*, was recently characterized and shown to be highly expressed during *in vivo *growth [74]. However, besides *AaSP2*, genes for secreted proteases of animal pathogenic oomycetes have not been reported. In this study, we identified a diverse set of 12 cDNAs of *S. parasitica *with similarity to the major catalytic classes of proteases. A number of the identified proteases had a signal peptide that would predict them to be localised at the interface between pathogen and host and suggests that they are candidate virulence factors.

Tian et al. [21] recently reported that plant pathogenic oomycetes secrete a diverse family of Kazal-like serine protease inhibitors with at least 35 members identified from *P. infestans*, *P. sojae*, *P. ramorum*, *P. brassicae*, and the downy mildew *Plasmopara halstedii*. Among these, the two-domain EPI1 protein and the three domain EPI10 of *P. infestans *were found to inhibit and interact with P69B, a defense subtilase of tomato, and were suggested to play a role in counterdefense [21]. Inhibitors of serine protease might be ubiquitous among eukaryotic parasites. For instance, the apicomplexan obligate parasite *Toxoplasma gondii *secretes TgPI-1 and TgPI-2, four-domain serine protease inhibitors of the Kazal family [75-77], and the intestinal hookworm *Ancylostoma ceylanicum *secretes an 8-kDa broad spectrum serine protease inhibitor of the Kunitz family [78]. Here we found that Kazal-like motifs also occur in Saprolegniales proteins.

In addition to Kazal-like motifs, we also discovered a cDNA that encodes a secreted protein with similarity to the cystatin class of cysteine protease inhibitors [79]. Cysteine protease inhibitors, such as chagasin, have been reported in animal parasites, mainly trypanosomids, and are thought to target proteases of the insect vector or the mammalian host [80-82]. Perhaps, inhibition of host proteases is a widespread counterdefense strategy in animal and plant pathogenic eukaryotes. Future studies will help to address whether the discovered protease inhibitors play a role in *S. parasitica*-fish interactions.

## Conclusion

This pilot cDNA sequencing project provides a first look into the gene content of *S. parasitica *and sets the basis for genomics research in this reemerging animal pathogen. Annotation of the ESTs revealed a number of genes that could function in virulence. Future work will focus on developing molecular tools for functional analysis of *S. parasitica *genes. In this regards, stable transformation of *Saprolegnia monoica *has been reported [83], and the RNAi protocol recently developed for *P. infestans *[84] should be adaptable to *S. parasitica*. Gene expression profiling will also be applied to investigate transcriptome changes during *S. parasitica*-fish interactions. Overall, these resources will greatly accelerate research on this important pathogen and could lead to novel perspectives for controlling saprolegniosis.

## Methods

### Strains and growth conditions

*Saprolegnia parasitica *ATCC90214, an isolate from lesions on coho salmon (*Oncorhynchus kisutch*) [25], was used in this study. Working stocks of this strain were routinely maintained on cornmeal agar (Difco Lab. Detroit, MI) at 18°C. To obtain axenically prepared mycelium, ATCC90214 was grown in GY broth (5 g glucose, 2.5 g yeast extract/L) for 29 days, which corresponds to stationary phase. Mycelium was harvested by filtration and immediately frozen prior to RNA extraction.

### cDNA construction

Total RNA from *S. parasitica *mycelium was isolated using the phenol-guanidine isothiocyanate based reagent Trizol, (Life Technologies Carlsbad, CA) according to the manufacturer's instructions. PolyA^+ ^mRNA was isolated using the oligotex mRNA purification kit (Qiagen, Valencia, CA). The cDNA library was synthesized and cloned in plasmid pSPORT1 using the Superscript™ plasmid system for cDNA synthesis and cloning (Invitrogen Life Technologies, Carlsbad, CA). Polyadenylated mRNA was used to synthesize oligo (dT) primed cDNAs, which were cloned unidirectionally in *Not*I/*Sal*I digested vector pSPORT1. Plasmid ligations were transformed into *Escherichia coli *ElectroMax-DH10B ™ cells (Invitrogen Life Technologies, Carsbad, CA). Selection was done on Luria-Bertani (LB) agar plates containing ampicillin (50 mg/L) [85]. Individual colonies were picked randomly with the Qpix robot (Genetix, Hampshire, UK) into 384 well plates containing LB freezing buffer (36 mM K_2_HPO_4_, 13.2 mM K_2_HPO_4_, 1.7 mM citrate, 0.4 mM MgSO_4_, 6.8 mM (NH4)_2_SO_4_, 4.4 % v/v glycerol in 1 × LB), incubated overnight without shaking, and stored at -80°C. Subsequently clones were transferred from the 384 well plate to 96 well plate for shipment to the Genomics Technology Support Facility (GTSF) at the Michigan State University where they were sequenced following manufacturer's recommendations using an ABI Prism 3700 DNA Analyzer. Identification codes for the cDNAs/ESTs were derived from the position of the corresponding cDNA clone in the microtiter plates preceded by SPM (for *Saprolegnia parasitica *mycelial) and the successive number of the microtiter plate.

### DNA sequencing

For the ESTs, DNA from bacterial cultures was purified at GTSF using Qiagen 3000 or Autogen 850 robots. Fluorescently labeled sequencing products were generated using the universal T7 primer resulting in 5' cDNA sequences. The sequencing products were separated by capillary electrophoresis on an ABI Prism 3700 DNA Analyzer (PE Applied Biosystems). A dataset representing 2296 EST sequences and the corresponding electropherograms were then made available through the Geospiza Finch web interface of GTSF. The complete inserts of selected cDNAs were sequenced by primer walking at the OARDC Molecular and Cellular Imaging Center (MCIC), Wooster, Ohio, using an ABI Prism 377 automated sequencer (PE Applied Biosystems).

### Bioinformatics

The sequences were processed using the XGI pipeline [28]. The assembly described in this paper is known as the May 2004 assembly. The consensus sequences (unigenes) were named Sp_N1_N2_May04 with N1 referring to the number of ESTs in the contig, and N2 the contig number. The consensus sequences were annotated using the methods implemented in the XGI pipeline [29]. These include BLASTX [86] searches against NCBI non-redundant (nr) protein library; BLIMPS search against Blocks+ protein motif database [87,88]; searches with the 12 algorithms of InterProScan [89] against the InterPro database [90]; and identification of signal peptides for extracellular secretion with PexFinder [91], an algorithm based on SignalP 2.0 [33,92]. Automated post-analysis annotation links BLAST and Blocks+ hits to their cognate Gene Ontology entries [93,94], whereas InterPro hits are automatically linked to GO annotations.

Additional similarity searches using BLAST [86] and other bioinformatics analyses were also performed locally on Mac OSX G4/G5 workstations. BLAST E-value lower than 0.01 were retained, and searches were conducted with the low-complexity filter on. Local databases were compiled from GenBank nonredundant (NR), dBEST, and TraceDB databases [95] and the Broad Institute [96]. They included "darwin_270334.faa" a curated dataset of 270334 eukaryotic proteins that we compiled. The data covers six major phyla: fungi, animals, plants, alveolates, discicristates, and heterokonts. It includes the complete proteomes of 17 species and at least the complete proteome of one species for all phyla except for discicristates. The MEROPS database of proteases and protease inhibitors was also queried [45]. Multiple alignments were conducted using the program Clustal-X [97], adjusted manually as necessary, and visualized with BOXSHADE [98]. Consensus sequences were visualized with weblogo [99]. A cDNA was deemed likely to be full length when it was the most 5' proximal EST among assemblies and gave hits to the N-terminal portion of known proteins following similarity searches.

### Phylogenetic analysis

A data set of concatenated protein sequences was developed to perform phylogenetic comparisons of four oomycete species and the diatom *Thalassosira pseudonana *[100]. First, BLASTX searches of the *S. parasitica *unigenes against the diatom proteome were performed. Matching sequences with E value < 1e-20 were extracted and then used to search WGS and EST reads of *P. infestans*, *P. sojae*, and *P. ramorum*. A total of 18 sequences that were conserved among all five species were identified and were aligned individually with Clustal-X. Poorly aligned edges were then trimmed and the alignments were concatenated. PAUP v4.0b8 (Sinauer Associates Inc., Sunderland, MA) was used to reconstruct phylogenetic trees using the neighbor joining method and maximum parsimony with 1000 bootstrap replications.

### Data dissemination

The DNA sequences, assemblies, and annotations are publicly available through the Oomycete Genomics Database (OGD) [1]. The 1510 high quality ESTs were also deposited in NCBI's GenBank under accession numbers DN615772-DN617281.

## Authors' contributions

TTA, performance of majority of wet lab experiments including construction of cDNA library, annotation and analyses of specific sequences, writing of manuscript. MT, sequencing of select cDNAs, annotation and analyses of specific sequences. KG, performance of bioinformatics analyses including OGD pipeline. MEW, performance of bioinformatics analyses including OGD pipeline, writing of manuscript. PvW, annotation and analyses of specific sequences, writing of manuscript. SK, supervision of experimental work, annotation and analyses of specific sequences, writing of manuscript.

## Figures and Tables

**Table 1:** Most represented protein domains in the *Saprolegnia parasitica *EST contigs

InterPro ID	Description	Number of contigs
IPR000719	Eukaryotic protein kinase	22
IPR004000	Actin and actin-like	11
IPR001993	Mitochondrial energy transfer proteins (carrier protein)	9
IPR000626	Ubiquitin domain	6
IPR002110	Ankyrin-repeat	5
IPR001680	G-protein beta WD-40 repeats	5
IPR000795	GTP-binding elongation factor	5
IPR001353	Multispecific proteasome proteases	5
IPR003008	Tubulin/FtsZ family	5
IPR000873	AMP-dependent synthetase and ligase	4
IPR000254	Cellulose-binding domain, fungal type	4
IPR002130	Cyclophilin-type peptidyl-prolyl cis-trans isomerase	4
IPR002048	EF-hand	4
IPR000173	Glyceraldehyde 3-phosphate dehydrogenase	4
IPR000232	Heat shock factor (HSF)-type DNA-binding domain	4
IPR001806	Ras GTPase superfamily	4
IPR000504	RNA-binding region RNP-1 (RNA recognition motif)	4
IPR001464	Annexin family	3
IPR001623	DnaJ N-terminal domain	3
IPR001753	Enoyl-CoA hydratase/isomerase	3
IPR001179	FKBP-type peptidyl-prolyl cis-trans isomerase (PPIase)	3
IPR001005	Myb DNA binding domain	3
IPR003014	PAN domain	3
IPR000668	Papain cysteine protease (C1) family	3
IPR001272	Phosphoenolpyruvate carboxykinase (ATP)	3
IPR001849	Pleckstrin homology (PH) domain	3
IPR001232	SKP1-like	3
IPR000063	Thioredoxin	3
IPR001440	TPR repeat	3
